# Validation of the PEN-FAST Score in a Pediatric Population

**DOI:** 10.1001/jamanetworkopen.2022.33703

**Published:** 2022-09-19

**Authors:** Ana Maria Copaescu, Sara Vogrin, Greg Shand, Moshe Ben-Shoshan, Jason A. Trubiano

**Affiliations:** 1 Department of Medicine, Division of Allergy and Clinical Immunology, McGill University Health Centre (MUHC), McGill University, Montreal, Quebec, Canada; 2 Department of Medicine, St Vincent’s Hospital, University of Melbourne, Fitzroy, Australia; 3 The Research Institute of the McGill University Health Centre, McGill University, MUHC, Montreal, Quebec, Canada; 4 Division of Allergy, Immunology and Dermatology, Montreal Children’s Hospital, MUHC, McGill University, Montreal, Quebec, Canada; 5 Centre for Antibiotic Allergy and Research, Department of Infectious Diseases, Austin Health, Heidelberg, Victoria, Australia

## Abstract

This cohort study examines a clinical decision model for penicillin allergies among pediatric patients; the model considers when reactions occurred; whether patients experienced angioedema, anaphylaxis, or a severe cutaneous adverse reaction; and whether treatment was required.

## Introduction

The pediatric prevalence of self-reported drug allergies is 10%,^1^ which carries significant health and economic implications.^2^ Following direct oral penicillin challenge, 94.6% of such labels are removed.^3,4^ However, despite published algorithms,^5^ there are no validated pediatric decision rules to guide clinician management. The aim of this cohort study was to examine the previously validated PEN-FAST adult score^6^ in children.

## Methods

Using a Canadian prospective pediatric cohort from 3 centers,^3^ we examined the PEN-FAST score in 2028 children with 2031 penicillin allergy labels (eMethods in the Supplement). Data were collected from August 8, 2011, to March 3, 2021. This cohort study was approved by the McGill University Ethics Committee and the Research Ethics Board at the University of Manitoba, and written informed consent was collected. Sample characteristics are presented as median (IQR) and frequency (%). The PEN-FAST score and area under the curve (AUC) were calculated. Logistic regression with components of the score was performed. Sensitivity analysis with different time categories and removal of severe cutaneous adverse reaction (SCAR) was performed, and subgroup analysis for immediate and delayed reactions and various age groups were performed. All analyses were performed in Stata version 16.1 (StataCorp).

## Results

The median (IQR) age for the 2028 children in the cohort was 4.3 (2.1-8.0) years, with mostly male participants (1091 [53.7%]). Most reported reactions occurred in the past 5 years or at an unknown time (1661 [81.8%]), with amoxicillin suspected in 2022 reactions (99.6%) (Table 1). Anaphylaxis and angioedema were reported in 229 cases (11.3%). Treatment (or unknown) was administered for 1231 cases (60.6%). The AUC for the PEN-FAST score was calculated at 0.528, showing poor discrimination ability. Using the published adult PEN-FAST cutoff of 3 or greater, the AUC was 0.510 (95% CI, 0.47-0.56), and sensitivity and specificity were 57.0% (95% CI, 47.1%-66.5%) and 45.7% (95% CI, 43.5%-48.0%), respectively. The negative predictive value was 95.0% (95% CI, 93.4%-96.3%), considered poor in the context of a low prevalence positive challenge (5%). Furthermore, none of the individual variables were associated with a positive test.

**Table 1.** Allergy Characteristics for the 2031 Allergy Labels in 2028 Participants

Characteristic	No. (%)
Age at adverse reaction, median (IQR), y	1.8 (1-3.7)
No.	1906
Time since reaction, median (IQR), y	1.2 (0.4-4.0)
No.	1946
Time since reaction	
Last year	879 (43.3)
1-5 y	697 (34.3)
More than 5 y	370 (18.2)
Unknown	85 (4.2)
Last 5 y or unknown^a^	1661 (81.8)
Reported drug exposure	
Amoxicillin	2022 (99.6)
Penicillin unspecified	8 (<1.0)
Penicillin V	1 (<1.0)
Patient-reported symptoms	
Pruritus, localized	59 (2.9)
Pruritus, generalized	523 (25.8)
Urticaria	1163 (57.3)
Flushing	381 (18.8)
Rhino conjunctivitis	35 (1.7)
Angioedema	227 (11.2)
Throat tightness	14 (0.7)
Stridor	6 (0.3)
Gastrointestinal	98 (4.8)
Breathing difficulties	38 (1.9)
Wheezing	15 (0.7)
Cyanosis	11 (0.5)
Circulatory collapse	1 (<1)
Hypotension	1 (<1)
Hypoxia	1 (<1)
Incontinence	2 (0.1)
Macular or papular rash	790 (38.9)
Erythema multiforme	72 (3.5)
Arthritis or arthralgia	91 (4.5)
Fever	114 (5.6)
Involvement of mucosal membranes	5 (0.2)
Other	173 (8.5)
Anaphylaxis, angioedema, or SCAR requiring treatment^a^	229 (11.3)
Epinephrine IM	14 (0.7)
Corticosteroids	74 (3.6)
Antihistamines	973 (47.9)
Anti-H2	8 (0.4)
IV fluids	15 (0.7)
Short-acting inhaled β agonists	7 (0.3)
Unknown	190 (9.4)
Treatment (any treatment or unknown)^a^	1231 (60.6)
Drug used for oral challenge	
Amoxicillin	2021 (99.5)
Amoxicillin/clavulanic acid	3 (<1)
Penicillin	5 (<1)
Result of skin prick test	
No.	1389
Positive	5 (0.2)
Negative	26 (1.3)
Not performed	1335 (65.7)
Unknown	23 (1.1)
Results of oral challenge	
Negative graded challenge	1924 (94.7)
Positive graded challenge	107 (5.3)

Abbreviations: IM, intramuscular; IV, intravenous; SCAR, severe cutaneous adverse reaction.

^a^ Individual variable of the PEN-FAST model, which was derived using an adult data set. The 4 features associated with a positive penicillin (PEN) allergy test result were F, 5 or fewer years since reaction; A, angioedema or anaphylaxis (adjudged by the clinician if the history was consistent with a cutaneous manifestation plus 1 respiratory, cardiovascular, or gastrointestinal symptom or acute onset hypotension or bronchospasm or airway obstruction alone); S, SCAR; and T, treatment required or unknown. A cutoff of less than 3 was previously used to define a low-risk penicillin allergy, with a negative predictive value of 96.3% (95% CI, 94.1%-97.8%).

Changing the coding for timing (<1 year) or removing the angioedema reported symptom did not improve the performance of the PEN-FAST tool in this pediatric population (Table 2). A subgroup analysis for the positive skin testing or challenges based on immediate vs delayed reaction or the time of the reported allergy showed similar results (Table 2). When the tool was used in children 13 years or older, the AUC was 0.622, indicating that despite variable adjustment, the tool is not useful (Table 2).

**Table 2.** Subgroup Analysis for Positive Penicillin Testing (Skin Testing or Challenge), According to Various Age Groups and Considering the Timing Since the Reaction

Subgroups	Participants, No.	AUC	Prevalence (95% CI), %	Sensitivity (95% CI), %	Specificity (95% CI), %	PPV (95% CI)	NPV (95% CI)
Immediate vs delayed positive reactions							
Immediate	48	0.524	2.4 (1.7-3.1)	45.8 (31.4-60.8)	45.4 (43.2-47.6)	2.0 (1.3-3.0)	97.2 (95.9-98.2)
Delayed	61	0.560	3.0 (2.3-3.8)	63.9 (50.6-75.8)	45.9 (43.7-48.1)	3.5 (2.5-4.8)	97.6 (96.4-98.5)
Age groups, y							
≤2	472	0.535	6.4 (4.3-9.0)	60.0 (40.6-77.3)	36.7 (32.1-41.3)	6.0 (3.6-9.4)	93.1 (88.3-96.4)
2-12	1339	0.533	4.9 (3.8-6.2)	57.6 (44.8-69.7)	45.1 (42.3-47.9)	5.2 (3.7-7.0)	95.3 (93.3-96.9)
≥13^a^	171	0.622	4.7 (2-9.0)	37.5 (8.5-75.5)	73 (65.5-79.7)	6.4 (1.3-17.5)	96 (90.8-98.7)
Timing of the reaction							
Within the last year	879	0.530	6.0 (4.5-7.8)	62.3 (47.9-75.2)	32.6 (29.4-35.9)	5.6 (3.9-7.8)	93.1 (89.5-95.7)
>1 y Before testing	1067	0.574	4.8 (3.6-6.2)	52.9 (38.5-67.1)	58.2 (55.1-61.2)	6.0 (4.0-8.6)	96.1 (94.2-97.5)

Abbreviations: AUC, area under the curve; NPV, negative predictive value; PPV, positive predictive value.

^a^ There were no patients aged 13 to 15 years old; therefore, aged 13 years or older represents participants aged 15 years or older.

## Discussion

In this Canadian pediatric prospective multicenter cohort, the PEN-FAST tool did not help identify low-risk penicillin allergies. This previously validated tool in an adult population was not useful for risk stratification in children younger than 12 years. In teenagers (≥13 years), the predictive ability of the tool increased (higher AUC, specificity, and NPV but lower sensitivity), indicating that the tool could have some value in this population following further study. The extrapolation of the results is limited by the small number of teenagers included.

The PEN-FAST performed similarly for immediate and delayed reactions, and the timing of the reported reaction was not associated with the outcome of the allergy investigation. In this context, it is possible that the low validity of the PEN-FAST tool in children is explained by the increased prevalence of viral-induced reactions compared with true drug hypersensitivity. This analysis adds to the evidence that true drug allergies are rare among children and that they are often incorrectly labeled during a viral infection. Furthermore, the criteria included in the PEN-FAST score might not provide adequate information considering different index reactions in the pediatric population compared with the adult population. This study highlights that children are not little adults and clinical decision rules need to be derived and validated in the target population.

These findings suggest that the PEN-FAST drug allergy clinical decision rule should not be adapted to a pediatric population younger than 12 years at this time. New validated point-of-care clinical tools are required to identify low-risk penicillin allergies in a pediatric population, and validation of PEN-FAST in adolescents requires further examination in extended international cohorts.

## Notes

Supplement.
eMethods.
