# Supplementary material for: Validation of the PEN-FAST Score in a Pediatric Population
Source: JAMA Netw Open. 2022 Sep 19;5(9):e2233703. doi: 10.1001/jamanetworkopen.2022.33703 (PMC9486451; doi:10.1001/jamanetworkopen.2022.33703)
Supplement: Supplement. — eMethods. [file jamanetwopen-e2233703-s001.pdf]

Supplemental Online Content

Copaescu AM, Vogrin S, Shand G, Ben-Shoshan M, Trubiano JA. Validation of the PEN-FAST score in a pediatric population. *JAMA Netw Open.* 2022;5(9):e2233703. doi:10.1001/jamanetworkopen.2022.33703

eMethods.

This supplemental material has been provided by the authors to give readers additional information about their work.

© 2022 Copaescu AM et al. *JAMA Network Open.*

eMethods

The study was approved by the McGill University Ethics Committee and the Research Ethics Board at the University of Manitoba. To participate, parents/legal guardian(s) signed the consent form on the behalf of children (<7 years old) and older children and adolescents cosigned with their parents/legal guardian.

Children were recruited from 3 centers: the Montreal Children's Hospital, an outpatient allergy clinic affiliated with the Montreal Children's Hospital, and the Meadowood Medical Centre, Winnipeg. The patients included had reported a history of a benign reaction to amoxicillin defined as a reaction limited to the skin with no mucosal involvement and absence of vesicles. The index reaction was retrospectively characterized but the patients were investigated prospectively.

PEN-FAST decision rule - The model was derived using backward stepwise logistic regression that included clinical variables predictive of a positive penicillin allergy test result, as previously published (Trubiano et al. 2020). In this study, the four features associated with a positive penicillin (PEN) allergy test result were: (F), five or fewer years; (A), angioedema/ anaphylaxis (adjudged by the clinician if the history was consistent with a cutaneous manifestation plus one of respiratory, cardiovascular or gastrointestinal symptoms or acute onset hypotension or bronchospasm/airway obstruction alone); (S), severe cutaneous adverse reaction (SCAR); and (T), treatment required or unknown. A cut-off of <3 was used to define a low risk penicillin allergy, with a NPV of 96.3% (95% CI, 94.1%-97.8%) (Trubiano et al. 2020).

All analyses were performed using *Stata 16.1*.

© 2022 Copaescu AM et al. *JAMA Network Open.*
